# Wounds of the Heart

**Published:** 1901-01-05

**Authors:** 


### WOUNDS OF THE HEART.

DR. L. L. HILL (New York *Medical Record*) records two cases of wound of the heart in which recovery took place. The first case was that of a child, aged eight years.

She had carried in her skirt waist a needle 2½ inches in length, which she had driven into her heart by accidentally falling against a tree. On being seen her countenance was anxious, the pulse was rapid and weak, and the respiration somewhat laboured. She had slight pain about her heart. On inspection it was found that the needle had entered the fifth intercostal space on the left side, and with the pulsations of the heart the head of the needle could be seen to move under the skin. Ten drops of a 4 per cent. solution of cocaine having been injected under the skin, an incision was made down to the needle, which was then extracted with a pair of dressing forceps. The wound was closed and there was no further trouble. The second case was that of a coloured man, aged 28 years, who had received a stab wound in the fourth intercostal space, a little to the right of the left nipple. Seen shortly after the injury there was complete relaxation of the extremities, the pulse was hardly perceptible, and the countenance indicated much distress. (In the flowery language with which American physicians love to embroider their medical records, "the countenance depicted a suffering and distress that would seem to welcome death with the wintry sterility of the grave as a kind and sympathising friend.") The heart sounds were very indistinct, there was increased cardiac dulness, and it was evident that the pericardium was rapidly filling with blood, and that the heart's action would soon be overcome by compression. The external hæmorrhage was slight. On evacuating the blood, however, by enlarging the opening in the pericardium, the patient's condition at once improved, and he ultimately recovered, notwithstanding that traumatic pericarditis supervened. Commenting upon these cases Dr. Hill says that no one will question the propriety of operative interference in wound of the heart, and he gives a *résumé* of seventeen recorded cases of heart suture. All operators are agreed, that interrupted sutures are to be preferred in closing a wound of the myocardium. They should be placed close together, and should not involve the endocardium. Silk is the best material to employ. The suture should, he says, be passed and tied during diastole, and the suture first inserted may be used to steady the heart during the passage of the others. In sewing the pericardium either interrupted or continuous sutures may be employed. For purposes of temporary hæmostasis some operators have recommended Péan's forceps, or their various modifications, but they all more or less lacerate the myocardium and can never replace digital pressure. Of course, such operations as are here mentioned involve the resection, or the turning back, of from one to three or four ribs.

